# The association of intrauterine and postnatal growth patterns and nutritional status with toddler body composition

**DOI:** 10.1186/s12887-023-04155-2

**Published:** 2023-07-06

**Authors:** Elizabeth Masiakwala, Lukhanyo H. Nyati, Shane A. Norris

**Affiliations:** 1 SAMRC/Wits Developmental Pathways for Health Research Unit, Department of Paediatrics, Faculty of Health Sciences, University of the Witwatersrand, 7 York Rd, Parktown, Johannesburg, 2193 South Africa; 2 Interprofessional Education Unit, Faculty of Community and Health Sciences, University of the Western Cape, Cape Town, South Africa; 3 School of Human Development and Health, University of Southampton, Southampton, UK

**Keywords:** Infant growth, Undernutrition, Paediatric obesity, Fat mass, Lean body mass

## Abstract

**Background:**

Growth patterns may be indicative of underlying changes in body composition. However, few studies have assessed the association of growth and body composition in poorly resourced regions experiencing the double-burden of malnutrition exists. Thus, the aims of this study were to investigate the association of intrauterine and postnatal growth patterns with infant body composition at 2 years in a middle-income country.

**Methods:**

Participants were from the International Atomic Energy Agency Multicentre Body Composition Reference study. Fat mass (FM), fat free mass (FFM), Fat mass index (FMI), fat free mass index (FFMI), and percentage fat mass (%FM) were measured in 113 infants (56 boys and 57 girls), from Soweto, South Africa, using deuterium dilution from 3 to 24 months. Birthweight categories were classified using the INTERGROWTH-21 standards as small (SGA), appropriate (AGA), and large-for gestational age (LGA). Stunting (> -2 SDS) was defined using the WHO child growth standards. Birthweight z-score, conditional relative weight and conditional length at 12 and 24 mo were regressed on body composition at 24 mo.

**Results:**

There were no sex differences in FM, FFM, FMI and FFMI between 3 and 24 mo. SGA and AGA both had significantly higher %FM than LGA at 12 mo. LGA had higher FM at 24 mo. Children with stunting had lower FM (Mean = 1.94, 95% CI; 1.63–2.31) and FFM (Mean = 5.91, 95% CI; 5.58–6.26) at 12 mo than non-stunting, while the reverse was true for FFMI (Mean = 13.3, 95% CI; 12.5–14.2) at 6 mo. Birthweight and conditionals explained over 70% of the variance in FM. CRW at both 12 and 24 mo was positively associated with FM and FMI. CRW at 12 mo was also positively associated with FMI, while CH at 24 mo was negatively associated with both FFMI and FMI in boys.

**Conclusion:**

Both LGA and SGA were associated with higher body fat suggesting that both are disadvantaged nutritional states, likely to increase the risk of obesity. Growth patterns through infancy and toddler period (1–2 years) are indicative of body fat, while growth patterns beyond infancy are less indicative of fat-free mass.

**Supplementary Information:**

The online version contains supplementary material available at 10.1186/s12887-023-04155-2.

## Introduction

Paediatric obesity is rising globally, and the prevalence is rising faster in low- and middle-income countries (LMIC) than other regions [1–4]. In 2020, 39 million children under the age of 5 had obesity or overweight with almost half of the number living in the developing countries [5]. By 2030, it is estimated that 28% of children between 5 and 9 years of age will have obesity in South Africa, which is higher than the estimated prevalence in the United States of America [6]. Obesity tracks through the life course, and infant weight and length gain are associated with lifelong overweight and obesity [7, 8].While sub-Saharan Africa (SSA) is experiencing the fastest rise in obesity [9], the prevalence of stunting has remained persistently high (~ 40%), leading to the existence of a double-burden of malnutrition [10, 11].

Anthropometric measurements such as the body mass index (BMI) are the most commonly used proxy for body composition, and growth patterns in utero and infancy play a key role in laying the foundation for excess fat accrual [12, 13]. However, BMI does not differentiate between the components of body composition [14]. It has been suggested that the acceleration in postnatal fat gain precedes the acceleration in body mass, subsequent to intrauterine growth restriction [15], leading to a mismatch between body fat and body size among malnourished children [16]. Others have shown that fat mass gain during infancy is a stronger predictor of the onset and risk of cardiometabolic disease in adulthood than rapid weight gain [17–19]. Thus, given the continued use of anthropometry, and the growing need to better characterise multicompartmental body composition, there is a need to examine the associations between growth and objectively measured body composition.

A number of studies have examined the association between growth and body composition [20–22], but there is scarcity of data in SSA. Assessing these patterns during critical periods of fat mass accrual may elucidate on the ability of growth to reflect changes in body composition. The period between birth and 6 months is considered a critical period, with babies experiencing a 30% increase total body fat increase by 30% in the first 6 months than at 2 years [23]. Thus, the aims of this longitudinal study of infants from a middle-income country were to assess (i) the relationship between intrauterine growth (using birthweight as a proxy) and postnatal changes in body composition, (ii) the relationship between postnatal nutritional status (stunting) and concurrent body composition, and (iii) the association between birthweight, growth (0–12 months and 12– 24 months) and body composition at 24 months.

## Methods and study design

### Study design

Data for this study was a secondary analysis of data drawn from the International Atomic Energy Agency Multicentre Body Composition Reference Study (IAEA-MBCRS). IAEA-MBCRS is a longitudinal study on singleton infants born in Soweto between 2014 and 2019. A total of 411 black mothers of infants born at Chris Hani Baragwanath Academic Hospital, South Africa, were recruited into the study. The commitment to the IAEA was to recruit 100 mother-infant pairs for inclusion into the global study. For the current study, a total of 115 infants with data at birth, 12 and 24 months were included. Sensitivity analyses were performed by comparing excluded participants to those who were included in the analytical sample. Mothers who were 18 years and above living in Soweto urban township, which is part of the Johannesburg Metropolitan Council Area in South Africa, were randomly approached and recruited at the maternity ward at Chris Hani Baragwanath Academic Hospital. Eligibility was based on the following criteria: (i) mothers had to be residents of Soweto, 18 years and above, must have delivered a full term (between 37- and 42-weeks) singleton healthy baby; (ii) A non-smoker and willing to breastfeed for up to 6 months and must have attained at least secondary school education level. Eligible mother-baby pairs were then followed up from birth until they reached 24 months. Infants with significant morbidity and congenital abnormalities were excluded from the study. All mothers provided written informed consent, and the ethical approval to conduct the study was granted by the University of the Witwatersrand Ethics Committee for Research on Human Subjects (M200997). All processes and methods were carried out in accordance with the IAEA-MBCRS guidelines and regulations.

### Anthropometric measurements

Anthropometric measurements were taken at 3, 6, 9, 12, 15, 18, 24 months follow-up visits as per World Health Organization (WHO) Multicentre Growth Reference Study (MGRS) standardized methodology [24, 25]. A portable electronic scale (Seca 376) placed on a flat surface and covered with a soft paper towel to protect the baby from direct contact with the cold surface was used to measure weight. The scale was then tared before placing a naked baby and measurement was recorded when the baby was calm. For measurement reliability, all infant measurements were taken in duplicate and recorded blinded by two research assistants. The supine length was measured from the crown of the head to the heels using a calibrated Harpenden Infantometer (Range 300-1100 mm) with digital counter readings to the nearest 0.1 cm. For measuring of length, the Infantometer with a perpendicular fixed headboard and a movable footboard was placed on a flat surface with a soft paper towel on top to prevent the baby from direct contact with the cold surface. The measurement was taken with the head touching an immovable heard board, positioned in the Frankfort vertical plane, secured by the supporting research assistant standing at the head position. The lead measurer stood on the side to hold the baby’s legs together making sure that the knees were straightened, and the footboard moved gently towards the heels of the baby. Quality control of the instruments was performed daily before use. Weighing scale (Seca 376) was calibrated using different weights of 0.5 kg, 1 kg, 2 kg, and 5 kg. Harpenden Infantometer was calibrated using two aluminium metallic rods of 75 cm and 40 cm.

### Derived variables

Birth size categories were classified as small for gestational age (SGA), appropriate for gestational age (AGA), and large for gestational age (LGA) using INTERGROWTH-21 standards [26]. Length-for-age (HAZ) was computed using 2006 WHO child growth standards for children from birth to five years. Stunting was defined as HAZ <-2 [27]. Conditional relative weight and conditional length) were obtained as standardised residuals by regressing previous length and weight on weight and length 12 and 24 months [28]. Conditional relative weight is the weight at current age accounting for current height and previous weight, while conditional length does not account for current weight [28]. For example, conditional relative weight at 24 months was obtained by regressing weight at birth and 12 months, and length at 12 and 24 months on weight at 24 months. Comparison by nutritional status (e.g., stunting vs. non-stunting) was assessed using the independent t-tests.

### Body composition measurement

Total body water (TBW) was measured by stable isotope dilution method using 99.8 atom % D, Sigma-Aldrich deuterium oxide isotope as outlined in the IAEA standard operating procedures [29]. Infants’ saliva samples were collected at 3, 6, 9, 12, 15, 18, and 24 months follow-up visits. Sample collection was a 3-hour process that was based in three stages: Baseline sample collection, dose administration and post dose collection. Baseline saliva sample was collected 20 min after the baby’s last feed by placing a ball of clean cotton wool inside the baby’s mouth until it was soaked wet. The soaked cotton wool was carefully removed and transferred into 20ml syringe. Saliva sample was then squeezed out into a 2ml vial labelled with a unique infant identification code. The baby was slowly given 1ml dose of undiluted deuterium oxide making sure that no spillages occurred. Another saliva sample was then collected 3 h post dose administration, assuming the enrichment distribution reached equilibrium. Samples were stored in -20 degrees Celsius until analysis.

Saliva samples were analysed in duplicate using portable 4500 Tumbl-IR Fourier transform infrared (FTIR) spectrometer (Agilent Technologies, CA, USA) according to manufactures instructions. The instrument is fitted with optics suitable for absorbance measurements on liquid samples in the mid-infrared range, and only takes about 20–30 µl of sample. To prevent cross-contamination between samples, the sampling window was cleaned with 99.9% ethanol after each analysis. Within day coefficient of variations were run daily before and after saliva analysis on the FTIR.

FFM was calculated from the measurement of TBW by the hydration factors [23]. FM was then derived as the difference of body weight and FFM and presented as kg or percentage. Body composition indices (FMI and FFMI) were defined as fat mass or lean mass in relation to height and weight. Log-log index (FM/FFM^*P*^) was calculated by performing a natural log transformation of FM and FFM then regressing log FM on log FFM. The regression coefficient was used as *P* in the ratio of absolute FM and FFM, as FM/FFM^*P*^. To present readable results, the derived index values were multiplied by 1000 [30].

### Data analysis

Continuous data were assessed for normality and where appropriate presented as means (95% CI) and as percentages for categorical variables. The independent t-test and Mann-Whitney U test were used to assess differences between groups for continuous normally and non-normally distributed variables from birth to 2 years, respectively. Differences between groups for categorical variables were compared using the Pearson chi-square test. The one-way analyses of variance (ANOVA), with Bonferroni multiple comparisons test, was used to assess differences in body composition between categories of birthweight (SGA, AGA, and LGA). FM, FMI, FFM, FFMI, and FM/FFM^*P*^ were each regressed on birthweight z-score, conditional relative weight, and conditional length at 12 months and 24 months using multiple linear regression analyses. All data were analysed using Stata 14.2 version (Stata-Corp LP, College Station, Texas, USA). Statistical differences were accepted at p < 0.05.

## Results

There were no significant differences in maternal demographic characteristics and infant anthropometric characteristics between included and excluded participants (Table S1).

### Maternal characteristics

Maternal characteristics for 115 infants (51% girls) are described in Table 1. The average age of the mothers was (26.6, 95% CI; 25.6–27.5) years and they had an average of (12.8, 95% CI; 11.0-14.5) years of schooling. Most of the mothers were not married (89.3% & 86.4% for boys and girls respectively), with about 12% being either married or cohabiting for both sexes. At screening, most mothers were primi- or multiparous, with 58.2% & 64.9% having at least one live-birth among the mothers of boys and girls respectively.

**Table 1:** Maternal characteristics stratified by child sex (boys and girls)

Maternal variable	Total	Boys (mean 95% CI)	Girls (mean 95% CI)	P value
N	115	56	59	
Age (years)	26.6 (25.6–27.5)	26.2 (24.6–27.8)	26.8 (25.6–27.5)	0.508
Gestational age (weeks)	39.3 (39.0-39.6)	39.3 (38.9–39.7)	39.3 (38.9–39.7)	0.945
Maternal education (years)	12.8 (11.0-14.5)	12.0 (11.9–12.0)	13.5 (10.0-14.5)	0.384
Parity (Freg, %)				
None	43 (38.4)	23 (41.8)	20 (35.1)	0.679
One	45 (40.2)	20 (36.4)	25 (43.9)	
Two+	24 (21.4)	12 (21.8)	12 (21.1)	
Marital status (Freq, %)				
Single/divorced/widowed	101 (87.8.6)	50 (89.3)	51 (86.4)	0.191
Married/cohabiting	14 (12.2)	6 (10.7)	8 (13.6)	

### Sex differences in anthropometric characteristics and body composition (Table S2)

There were no sex differences in FM, FFM, FMI, FFMI, and FM/FFM between 3 and 24 months.

### Association between size at birth and body composition

**Fig. 1:**
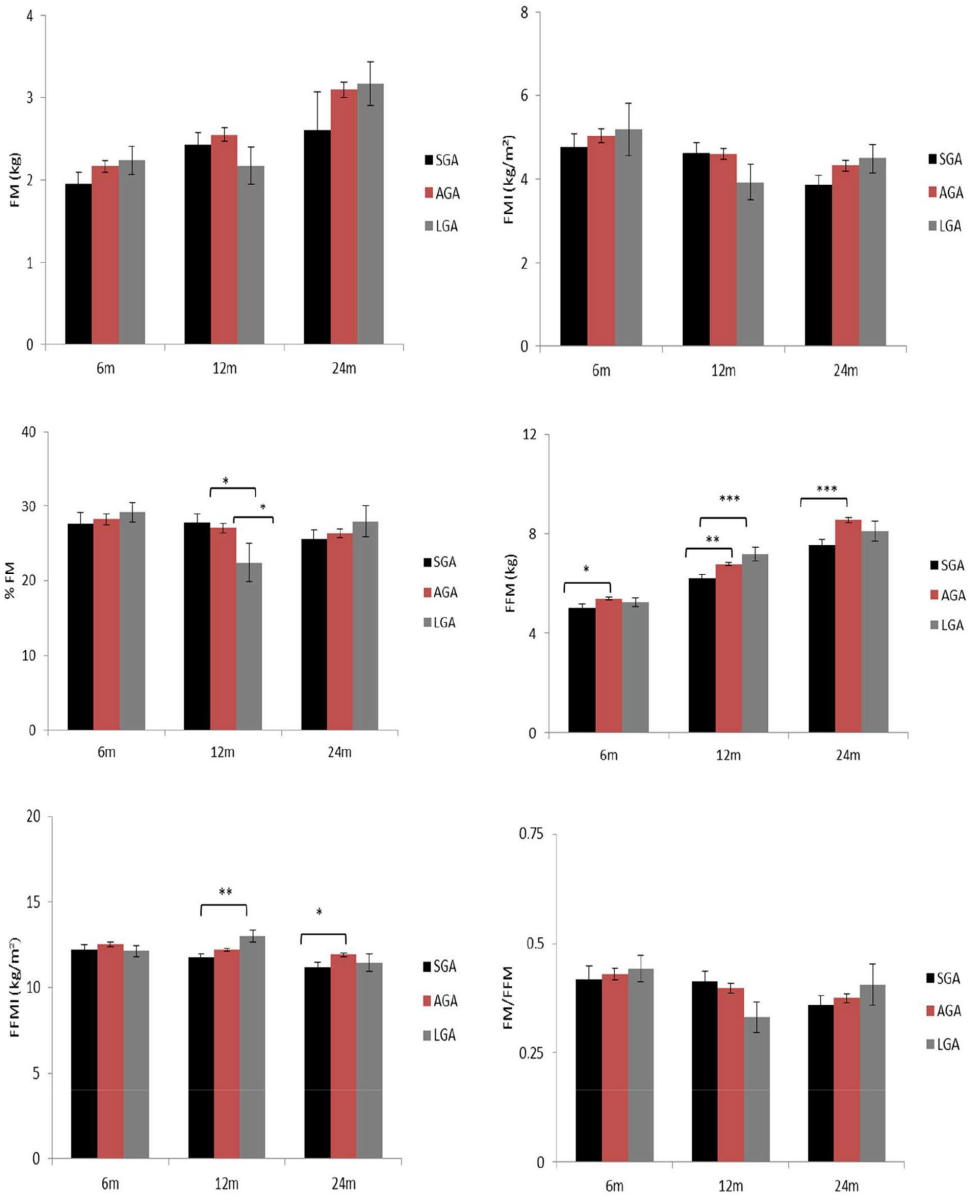
Relationship between birthweight categories at 6, 12, and 24 months with body composition at 2 years. There were no significant differences in FM and FMI between categories of birth weight. At 12 months, both small for gestational age (SGA) and appropriate for gestational age (AGA) had significantly higher %FM than large for gestational age (LGA) (p < 0.05). AGA had higher FFM than SGA at 6 (p < 0.05), 12 (p < 0.01) and 24 (p < 0.001) months. Additionally, LGA had higher FFM (p < 0.001) than SGA, at 12 months. Similarly, LGA had higher FFMI (p < 0.01) at 12 months than SGA, while AGA had higher FFMI (p < 0.05) at 24 months than SGA. Standard errors in parentheses: *** p < 0.001, ** p < 0.01, * p < 0.05

The association of birthweight categories and body composition at 6, 12 and 24 mo is described in Fig. 1. There were no significant differences in FM and FMI between categories of birthweight. At 12 mo, both (SGA) and (AGA) had significantly higher %FM than (LGA) (SGA 28.6, 95% CI; 26.4–30.8, AGA 27.9, 95% CI; 26.9–28.9, LGA 24.0, 95% CI; 19.6–28.3). AGA had higher FFM than SGA at 6 mo (SGA 5.0, 95% CI; 4.6–5.4, AGA 5.4, 95% CI; 5.2–5.6), 12 mo (SGA 6.2, 95% CI; 6.0-6.5, AGA 6.8, 95% CI; 6.7–6.9) and 24 mo (SGA 7.6, 95% CI; 7.1–8.1, AGA 8.5, 95% CI; 8.4–8.8)) mo. Additionally, LGA had higher FFM (7.2, 95% CI; 6.6–7.9) than SGA (6.3, 95% CI; 6.0-6.5), at 12 mo. Similarly, LGA had higher FFMI (13.1, 95% CI; 12.3–13.8)) at 12 mo than SGA (11.8, 9, 5% CI; 11.4–12.1), while AGA (11.9, 95% CI; 11.7–12.2) had higher FFMI ) at 24 mo than SGA (11.2, 95% CI; 10.6–11.6).

### Association of nutritional status with body composition

**Fig. 2:**
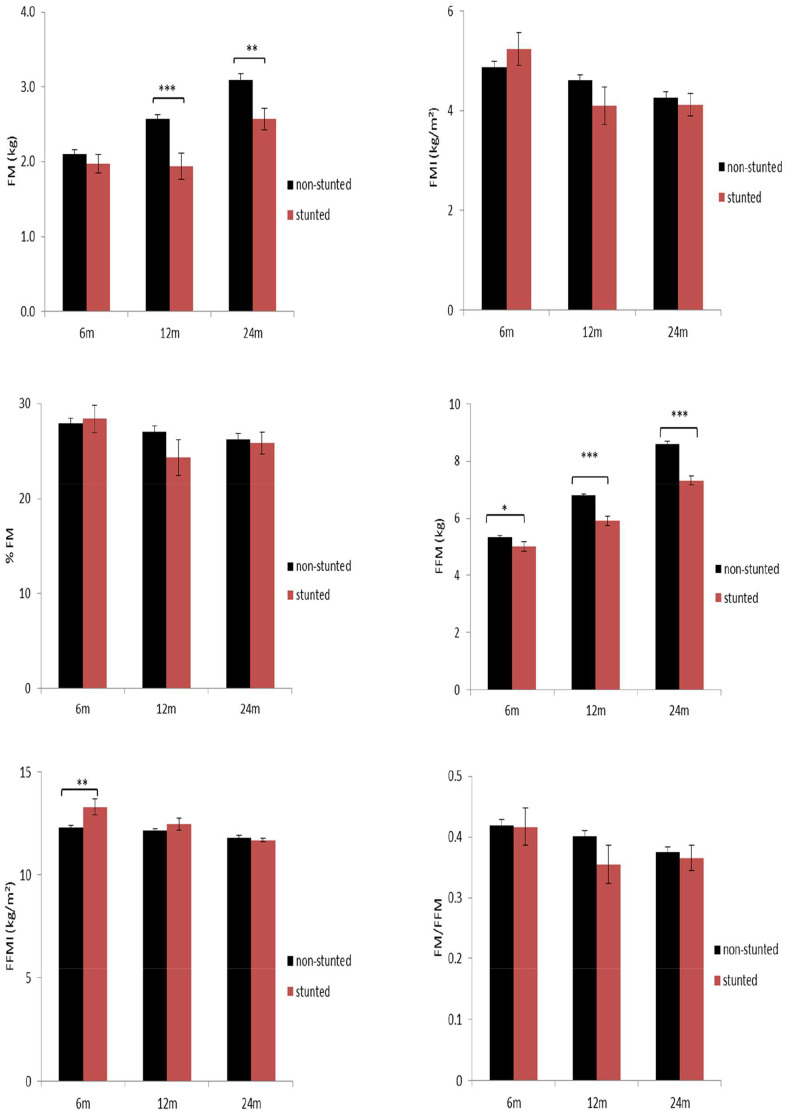
Relationship between nutritional status at 6, 12, and 24 months with body composition at 2 years. *** p < 0.001, ** p < 0.01, * p < 0.05

Associations between stunting and body composition at 6, 12, and 24 mo are described in Fig. 2. Children with stunting had lower FM (1.9, 95% CI; 1.6–2.3), (2.6, 95% CI; 2.3–2.9) and FFM (5.9, 95% CI; 5.6–6.3), 7.5, 95% CI; (7.0-7.7) at 12 and 24 mo and additionally at 6 mo for FFM (5.0, 95% CI; 4.7–5.4) than children with no stunting (FM at 12 & 24 mo (2.6, 95% CI; 2.5–2.7 & 3.1, 95% CI; 2.9–3.3), FFM at 12 & 24 mo (6.8, 95% CI; 6.7–6.9), 8.6, 95% CI; 8.4–8.8), FFM at 6 mo 5.3, 95% CI; 5.2–5.4). Conversely, children with stunting had higher FFMI (Mean 13.3, 95% CI; 12.5–14.2)at 6 mo than children without stunting (12.3, 95% CI; 12.1–12.5).

### Association of birthweight, conditional relative weight, and conditional length with body composition

Association of birthweight, conditional relative weight, and conditional length with body composition is described in Table 2. Birthweight was significantly associated with FFM (β (± SE) boys: 0.23 (-0.1), girls: 0.21 (-0.05). Conditional relative weight gain at 12 mo was positively associated with FM (boys: 0.64 (-0.08), girls: 0.31 (-0.06), FFM (boys: 0.49 (-0.06), girls: 0.37 (-0.06)), FMI (boys: 0.73 (-0.1), girls: 0.44 (0.1)), and FFMI (boys: 0.40 (-0.13), girls: 0.56 (-0.13). Conditional length at 12 mo was associated with FM (boys: 0.24 (-0.08), girls: 0.18 (-0.07) and FFM (boys: 0.59 (-0.08), girls: 0.59 (-0.07). Conditional length at 24 mo was positively associated with FM (0.22 (-0.08)) and FFM (0.29 (-0.08)) in girls, but negatively associated with FMI (-0.34 (-0.11) and FFMI (-0.38 (-0.14) in boys. Conditional weight at both 0-12mo (0.07 (0.02), (0.05 (0.02)) and 12-24mo (0.06 (0.02), 0.09 (0.02)) were associated with FM/FFMP in both boys and girls, respectively.

**Table 2:** The relationship between Birthweight z-score, conditional relative weight, and conditional length at 0–12 and 12–24 months with FM, FMI, FFM and FFMI at 24 months

Variables	FM (kg) β (± SE)		FFM (kg) β (± SE)		FMI (kg/m²) β (± SE)		FFMI (kg/m²) β (± SE)		FM/FFM^*p*^ β (± SE)	
N = 113	Boys	Girls	Boys	Girls	Boys	Girls	Boys	Girls	Boys	Girls
Birthweight z-score	0.08 (-0.1)	0.09 (-0.05)	0.23* (-0.1)	0.12* (-0.05)	0.07 (-0.13)	0.09 (-0.07)	0.14 (-0.16)	0.06 (-0.09)	0.05 (0.02)	0.02 (0.01)
Conditional relative weight (0–12 months)	0.64*** (-0.08)	0.31*** (-0.06)	0.49*** (-0.08)	0.37*** (-0.06)	0.73*** (-0.1)	0.44*** (-0.1)	0.40** (-0.13)	0.56*** (-0.13)	0.07*** (0.02)	0.05*** (0.02)
Conditional length (0–12 months)	0.24** (-0.08)	0.18* (-0.07)	0.59*** (-0.08)	0.59*** (-0.07)	0.05 (-0.1)	-0.02 (-0.11)	0.08 (-0.12)	0.15 (-0.14)	0.02 (0.02)	0.02 (0.02)
Conditional relative weight (12–24 months)	0.45*** (-0.09)	0.49*** (-0.07)	0.19* (-0.09)	0.32*** (-0.07)	0.56*** (-0.11)	0.61*** (-0.1)	0.24 (-0.14)	0.16 (-0.13)	0.06** (0.02)	0.09*** (0.02)
Conditional length (12–24 months)	-0.11 (-0.09)	0.22** (-0.08)	0.18 (-0.09)	0.29*** (-0.08)	-0.34** (-0.11)	0.04 (-0.12)	-0.38** (-0.14)	-0.27 (-0.15)	-0.02 (0.02)	0.04 (0.02)
R-squared	0.73	0.67	0.71	0.77	0.7	0.52	0.36	0.38	0.50	0.50

Abbreviations: FM, Fat Mass; FFM, Fat-Free Mass; FMI, Fat Mass Index; FFMI, Fat-Free Mass Index

Standard errors in parentheses: *** p < 0.001, ** p < 0.01, * p < 0.05

## Discussion

This study investigated the association of intrauterine growth, postnatal infant growth, and nutritional status with infant body composition at 2 years. Our findings revealed that there were significant differences between categories of birthweight and levels of nutritional status. Children born SGA and AGA both had significantly higher %FM than children born LGA at 12 months of age. Conversely, children born LGA had higher FFM and FFMI than SGA at 12 months. Additionally, AGA had higher FFM at 12 and 24 months and higher FFMI at 24 months than SGA. Stunting was associated with lower FM and FFM at 12 and 24 months and additionally at 6 months for FFM. Conversely, children with stunting had higher FFMI at 6 months than children with no stunting. Growth patterns from birth to 24 months were significantly associated with body composition at 24 months.

SGA is a proxy for intrauterine growth restriction, which may adversely influence fat metabolism. It has been suggested that there is a differential rate of growth between body size and composition among SGA, such that the acceleration in subcutaneous fat deposition precedes acceleration in weight during catch-up growth [31]. Children born SGA have a tendency towards higher fat absorption than AGA [32]. Consequently, SGA may have a greater propensity to accruing body fat [33], especially in abdominal depots [34], which may increase the risk of later cardiometabolic disease. Conversely, being LGA is considered a marker of in utero overnutrition, which is likely to predispose children to postnatal obesity [35]. Our findings also confirmed that SGA and LGA are both disadvantaged nutritional states in infancy, likely to increase the risk for obesity. We showed that SGA had higher %FM at 12 months than LGA, suggestive of increased risk of developing adiposity in SGA However %FM has limitations in that it does not account for body size when assessing adiposity in infants [36]. A Swedish study reported that both term SGA and LGA infants had higher percentage body fat than AGA in the first 4 months of life [37]. Hediger et al. found that differences in body fat between SGA and LGA only emerged at 6 years of age, with LGA having higher body fat at this age [38]. We found that AGA had higher FFM and FFMI at 24 months than SGA, affirming the metabolic advantage of this group.

In contrast to intrauterine growth restriction, postnatal nutritional deprivation was not associated with higher body fat. It has been suggested that during nutritional deprivation in infancy, FM may be preserved at the expense of length for optimum metabolic function [39]. To this end, it would be expected that children with stunting would have higher FM than those without stunting. We found that children with stunting had lower FM and FFM than children with no stunting. Surprisingly, infants with stunting in the present study had significantly higher FFMI at 6 months compared to children with no stunting. Others found that stunting was not associated with FFMI at 6 months [40], while stunting at 2 years was negatively associated with total fat-free soft tissue mass at 22 years [41].

Early infancy is a critical window for the development of adiposity early in life. Previous studies reported that weight gain in the first 6 months of life was a better predictor of fat mass later in life, than did after 6 months [42, 43]. We could not establish that rapid growth at 0–12 months was a better predictor of FM than growth at 12–24 months. Our findings in girls also showed that rapid linear growth was associated with greater FM and FFM at 24 months. These findings may highlight the effect of adiposity on linear growth during childhood. Forbes et al. hypothesised that childhood adiposity accelerates linear growth; meaning that in childhood heavier children are generally taller than their peers [44]. Similarly, a study reported an association between rapid linear growth in infancy with increased visceral adipose tissues at 22 years [41]. It has been shown that FM/FFM^*P*^ is statistically robust index which is highly sensitive to rapid changes in critical periods of infant growth due to the fact that absolute values of FM cannot explain interindividual fatness variations [45]. However, our findings showed that rapid weight gain was highly associated with FM, FFM, FMI, and FM/FFM^*P*^ at 0–24 months critical growth period.

This was the first longitudinal study to investigate the effect of growth patterns on nutritional status and body composition from birth to 2 years using the deuterium dilution method in our setting. Our data were from healthy breastfed infants of healthy educated mothers who were within the upper socioeconomic status. One limitation was that we did not have enough data on maternal pre-pregnancy BMI status which would have shed a light on whether LGA and high birthweight infants were born of women with obesity. The second limitation was that our findings were not representative of all infants and were biased to urban African infants. Due to our small sample size stunting and size at birth categories could not be stratified by sex. Lastly, this study did not adjust for feeding patterns to assess the association with body composition. Nonetheless, this provides the groundwork for subsequent studies on infant body composition in LMIC, particularly SSA. Understanding how growth patterns influence body composition in LMICs undergoing dual burden of malnutrition is pivotal.

## Conclusion

Although birthweight categories on their own showed that SGA at 12 months was associated with increased percentage fat mass, adjusting for postnatal growth patterns showed that it was conditional relative weight from 0–12 and 12–24 months that were positively associated with infant FM and FFM. Therefore, this suggests that postnatal catch-up growth may be more critical in influencing infant body composition than being born small for gestational age. There is a need for early intervention and monitoring of rapid gain patterns in infancy.

## Electronic supplementary material

Below is the link to the electronic supplementary material.

Supplementary Material 1

Supplementary Material 2

## Data Availability

All data collected, generated or analysed to support the findings of this study are available at the MRC/Wits Developmental Pathways for Health Research Unit, University of the Witwatersrand. However, data are available from the authors with the permission from the University of the Witwatersrand. For materials and data contact Elizabeth Masiakwala at masiakwalae@gmail.com.

## References

[1] Wang Y, Lobstein T (2006). Worldwide trends in childhood overweight and obesity. Int J Pediatr Obes.

[2] Popkin BM, Slining MM. New dynamics in global obesity facing low- and middle-income countries. Obes Rev. 2013. 10.1111/obr.12102 PMC4074506 24102717

[3] Tzioumis E, Kay MC, Bentley ME, Adair LS. Prevalence and trends in the childhood dual burden of malnutrition in low- and middle-income countries, 1990–2012. Public Health Nutr. 2016. 10.1017/S1368980016000276 PMC5470367 26905921

[4] Di Cesare M, Sorić M, Bovet P, Miranda JJ, Bhutta Z, Stevens GA et al. The epidemiological burden of obesity in childhood: a worldwide epidemic requiring urgent action. BMC Med. 2019. 10.1186/s12916-019-1449-8 PMC6876113 31760948

[5] World Health Organization. Obesity and overweight. Fact sheet. Updated 09 June 2021. WHO; 2021.

[6] Tim Lobstein and Hannah Brinsden. Atlas of Childhood Obesity. www.worldobesity.org (Access date 20/11/2021). 2019.

[7] Simmonds M, Burch J, Llewellyn A, Griffiths C, Yang H, Owen C (2015). The use of measures of obesity in childhood for predicting obesity and the development of obesity-related diseases in adulthood: a systematic review and meta-analysis. Health Technol Assess (Rockv).

[8] Gibson LY, Allen KL, Davis E, Blair E, Zubrick SR, Byrne SM (2017). The psychosocial burden of childhood overweight and obesity: evidence for persisting difficulties in boys and girls. Eur J Pediatr.

[9] Danquah FI, Ansu-Mensah M, Bawontuo V, Yeboah M, Kuupiel D (2020). Prevalence, incidence, and trends of childhood overweight/obesity in Sub-Saharan Africa: a systematic scoping review. Arch Public Heal.

[10] Said-Mohamed R, Micklesfield LK, Pettifor JM, Norris SA (2015). Has the prevalence of stunting in south african children changed in 40 years? A systematic review. BMC Public Health.

[11] Nyati LH, Pettifor JM, Norris SA (2019). The prevalence of malnutrition and growth percentiles for urban south african children. BMC Public Health.

[12] Poddar M, Chetty Y, Chetty VT (2017). How does obesity affect the endocrine system? A narrative review. Clin Obes.

[13] Hull HR, Herman A, Gibbs H, Gajewski B, Krase K, Carlson SE (2020). The effect of high dietary fiber intake on gestational weight gain, fat accrual, and postpartum weight retention: a randomized clinical trial. BMC Pregnancy Childbirth.

[14] Weber DR, Moore R, Leonard MB, Zemel BS (2013). Fat and lean BMI reference curves in children and adolescents and their utility in identifying excess adiposity compared with BMI and percentage body fat. Am J Clin Nutr.

[15] Gluckman PD, Hanson MA, Buklijas T (2010). A conceptual framework for the developmental origins of health and disease. J Dev Orig Health Dis.

[16] Gluckman PD, Hanson MA, Spencer HG (2005). Predictive adaptive responses and human evolution. Trends Ecol Evol.

[17] Araújo J, Ramos E (2017). Paediatric obesity and cardiovascular risk factors – A life course approach. Porto Biomed J.

[18] Howe LD, Chaturvedi N, Lawlor DA, Ferreira DLS, Fraser A, Davey Smith G (2014). Rapid increases in infant adiposity and overweight/obesity in childhood are associated with higher central and brachial blood pressure in early adulthood. J Hypertens.

[19] Munthali RJ, Kagura J, Lombard Z, Norris SA (2016). Childhood adiposity trajectories are associated with late adolescent blood pressure: birth to twenty cohort. BMC Public Health.

[20] Pereira-Freire JA, Lemos JO, de Sousa AF, Meneses CC, Rondó PHC (2015). Association between weight at birth and body composition in childhood: a brazilian cohort study. Early Hum Dev.

[21] Aneesh M, Ghugre PS (2019). Anthropometry, body fat and central adiposity in LBW and NBW indian children aged 3.5 to 4 years. Early Hum Dev.

[22] Gätjens I, Fedde S, Schmidt SCE, Hasler M, Plachta-Danielzik S, Müller MJ et al. Relationship between Birth Weight, early growth rate, and body composition in 5-to 7-Year-old children. Obes Facts. 2022;:1–9. 10.1159/000522509 PMC9421709 35292608

[23] Fomon SJ, Haschke F, Ziegler EE, Nelson SE (1982). Body composition of reference children from birth to age 10 years. Am J Clin Nutr.

[24] de Onis M, Garza C, Victora CG, Onyango AW, Frongillo EA, Martines J (2004). The WHO Multicentre Growth Reference Study: planning, study design, and methodology. Food Nutr Bull.

[25] De Onis M (2006). Reliability of anthropometric measurements in the WHO Multicentre Growth Reference Study. Acta Paediatr Int J Paediatr.

[26] Villar J, Ismail LC, Victora CG, Ohuma EO, Bertino E, Altman DG (2014). International standards for newborn weight, length, and head circumference by gestational age and sex: the newborn cross-sectional study of the INTERGROWTH-21st Project. Lancet.

[27] WHO Multicentre Growth Reference Study Group. WHO Child Growth Standards: Length/height-for-age, weight-for-age, weight-for-length, weight-forheight and body mass index-for-age: methods and development. Geneva, Switzerland. Acta Pædiatrica. 2006.

[28] Adair LS, Fall CHD, Osmond C, Stein AD, Martorell R, Ramirez-Zea M (2013). Associations of linear growth and relative weight gain during early life with adult health and human capital in countries of low and middle income: findings from five birth cohort studies. Lancet.

[29] IAEA. Introduction to body composition Assessment using the deuterium dilution technique with analysis of urine samples by isotope ratio Mass Spectrometry. IAEA Hum Heal Ser no 13. 2011.

[30] Abreu LRS, Shirley MK, Castro NP, Euclydes VV, Bergamaschi DP, Luzia LA (2019). Gestational diabetes mellitus, pre-pregnancy body mass index, and gestational weight gain as risk factors for increased fat mass in brazilian newborns. PLoS ONE.

[31] Okada T, Takahashi S, Nagano N, Yoshikawa K, Usukura Y, Hosono S (2015). Early postnatal alteration of body composition in preterm and small-for-gestational-age infants: implications of catch-up fat. Pediatr Res.

[32] Picaud J-C, Putet G, Rigo J, Salle B, Senterre J (1994). Metabolic and energy balance in small‐ and appropriate‐for‐gestational‐age, very low‐birth‐weight infants. Acta Pædiatrica.

[33] Cho WK, Suh BK. Catch-up growth and catch-up fat in children born small for gestational age. Korean J Pediatr. 2016. 10.3345/kjp.2016.59.1.1 PMC4753194 26893597

[34] Cho K, Suh B-K (2016). Catch-up growth and catch-up fat in children born small for gestational age. Korean J Pediatr.

[35] Lawlor DA, The society for social medicine John Pemberton Lecture. 2011. Developmental overnutrition-an old hypothesis with new importance. International Journal of Epidemiology. 2013. 10.1093/ije/dys209 23508404

[36] Wells JCK, Cole TJ (2002). Adjustment of fat-free mass and fat mass for height in children aged 8 y. Int J Obes.

[37] Larsson A, Ottosson P, Törnqvist C, Olhager E (2019). Body composition and growth in full-term small for gestational age and large for gestational age swedish infants assessed with air displacement plethysmography at birth and at 3–4 months of age. PLoS ONE.

[38] Hediger ML, Overpeck MD, McGlynn A, Kuczmarski RJ, Maurer KR, Davis WW (1999). Growth and fatness at three to six years of age of children born small- or large-for-gestational age. Pediatrics.

[39] Wells JCK, Chomtho S, Fewtrell MS (2007). Programming of body composition by early growth and nutrition. Proc Nutr Soc.

[40] Skau JKH, Grenov B, Chamnan C, Chea M, Wieringa FT, Dijkhuizen MA (2019). Stunting, wasting and breast-feeding as correlates of body composition in cambodian children at 6 and 15 months of age. Br J Nutr.

[41] Prioreschi A, Munthali RJ, Kagura J, Said-Mohamed R, Rolfe EDL, Micklesfield LK (2018). The associations between adult body composition and abdominal adiposity outcomes, and relative weight gain and linear growth from birth to age 22 in the birth to twenty plus cohort, South Africa. PLoS ONE.

[42] Sachdev HS, Fall CHD, Osmond C, Lakshmy R, Biswas SKD, Leary SD (2005). Anthropometric indicators of body composition in young adults: relation to size at birth and serial measurements of body mass index in childhood in the New Delhi birth cohort. Am J Clin Nutr.

[43] Botton J, Heude B, Maccario J, Ducimetière P, Charles MA, Basdevant A (2008). Postnatal weight and height growth velocities at different ages between birth and 5 y and body composition in adolescent boys and girls. Am J Clin Nutr.

[44] Forbes GB. Lean body mass and fat in obese children. Pediatrics. 1964. 14211097

[45] Herath MP, Ahuja KDK, Beckett JM, Jayasinghe S, Byrne NM, Hills AP (2021). Determinants of infant adiposity across the first 6 months of life: evidence from the baby-bod study. J Clin Med.

