# Supplementary material for: The association of intrauterine and postnatal growth patterns and nutritional status with toddler body composition
Source: BMC Pediatr. 2023 Jul 6;23:342. doi: 10.1186/s12887-023-04155-2 (PMC10324124; doi:10.1186/s12887-023-04155-2)
Supplement: Supplementary file 1 — Supplementary Material 1 [file 12887_2023_4155_MOESM1_ESM.docx]

**Table S1: Comparison of maternal characteristics and infant anthropometric measurements of total participants recruited and sub-sample.**

|  | Excluded Participants  Mean (95% Cl) | Included Participants  Mean (95% Cl) | P-values |
| --- | --- | --- | --- |
| N | Total number of participants | Total number of participants |  |
| Maternal Age (years) | 26.2 (25.6-26.8) | 26.8 (25.8-27.8) | 0.343 |
| Maternal Education (years) | 12.0 (11.9-12.2) | 12.8 (11.0-14.5) | 0.207 |
| Parity (%) |  |  |  |
| No child  1 child  2+ children | Frequency (42.5) | Frequency (35.1) | 0.497 |
|  | Frequency (34.7) | Frequency (43.2) |  |
|  | Frequency (22.8) | Frequency (21.6) |  |
| Gestational age (weeks) | 38.8 (38.3-39.2) | 39.3 (39.0-39.5) | 0.203 |
| Infant weight (kg) | | | |
| Birth | 3.2 (3.2-3.3) | 3.2 (3.1-3.3) | 0.722 |
| 12 mo | 9.4 (9.2-9.6) | 9.4 (9.2-9.7) | 0.762 |
| 24 mo | 11.6 (11.1-12.0) | 11.6 (11.3-11.9) | 0.960 |
| Infant length (cm) | | | |
| Birth | 50.6 (50.3-50.9) | 50.4 (49.8-50.9) | 0.454 |
| 12 mo | 74.3 (73.5-74.9) | 74.2 (73.5-74.9) | 0.831 |
| 24 mo | 84.4 (83.3-85.5) | 84.2 (83.5-84.9) | 0.756 |
