# Supplementary material for: The association of intrauterine and postnatal growth patterns and nutritional status with toddler body composition
Source: BMC Pediatr. 2023 Jul 6;23:342. doi: 10.1186/s12887-023-04155-2 (PMC10324124; doi:10.1186/s12887-023-04155-2)
Supplement: Supplementary file 2 — Supplementary Material 2 [file 12887_2023_4155_MOESM2_ESM.docx]

Supplementary Table

**Table S2: Body composition from 3-24 months by deuterium oxide dilution method**

|  | FFM (kg) | | FM (kg) | | FM (%) | | FFMI (kg/m^2^) | | FMI (kg/m^2^) | | FFM/FM | |
| --- | --- | --- | --- | --- | --- | --- | --- | --- | --- | --- | --- | --- |
|  | boys | girls | boys | girls | boys | girls | boys | girls | boys | girls | boys | girls |
| 3 months | 4.6 (0.6) | 4.3  (0.6) | 1.7  (0.5) | 1.7  (0.5) | 26.6  (5.9) | 28.1  (6.6) | 13.3  (2.5) | 12.5  (2.0) | 4.9  (1.6) | 4.9  (1.5) | 0.4  (0.1)*** | 0.4  (0.1) |
| 6 months | 5.7  (0.7) | 5.1  (0.7) | 2.2  (0.7) | 2.3  (0.7) | 27.2  (7.0) | 30.7  (7.3) | 12.9  (1.8) | 12.2  (1.7) | 5.0  (1.5) | 5.5  (1.7) | 0.4  (0.1)*** | 0.5  (0.2) |
| 9 months | 6.3  (0.8) | 5.8  (0.8) | 2.7  (0.8) | 2.6  (0.7) | 29.3  (6.7) | 30.5  (6.6) | 12.5  (1.6) | 11.8  (1.6) | 5.2  (1.5) | 5.3  (1.5) | 0.4  (0.1)*** | 0.5  (0.1) |
| 12 months | 7.0  (0.9) | 6.4  (0.8) | 2.7  (0.8) | 2.6  (0.9) | 27.3  (6.3) | 28.6  (7.4) | 12.7  (3.1) | 11.8  (1.6) | 4.8  (1.5) | 4.8  (1.6) | 0.4  (0.1)*** | 0.4  (0.2) |
| 15 months | 7.6  (1.0) | 7.1  (0.9) | 2.8  (0.8) | 2.9  (1.0) | 26.6  (5.3) | 28.9  (7.3) | 12.5  (1.1) | 11.8  (1.1) | 4.6  (1.2) | 4.9  (1.7) | 0.4  (0.1)*** | 0.4  (0.2) |
| 18 months | 7.9  (1.1) | 7.3  (0.9) | 2.8  (1.1) | 2.9  (0.7) | 25.8  (7.0) | 28.3  (5.5) | 12.6  (1.5) | 11.7  (1.3) | 4.4  (1.5) | 4.7  (1.1) | 0.4  (0.1)*** | 0.4  (0.1) |
| 24 months | 8.8  (1.1) | 8.1  (1.0) | 3.2  (1.1) | 3.2  (0.9) | 26.2  (5.9) | 27.9  (5.1) | 12.1  (1.3) | 11.7  (1.2) | 4.4  (1.4) | 4.5  (1.1) | 0.4  (0.1)*** | 0.4  (0.1) |

**P values: *p<0.05 **p<0.01 ***p<0.001**
